# Trends in birth weight and the prevalence of low birth weight and small-for-gestational-age in Surinamese South Asian babies since 1974: cross-sectional study of three birth cohorts

**DOI:** 10.1186/1471-2458-13-931

**Published:** 2013-10-07

**Authors:** Jeroen A de Wilde, Stef van Buuren, Barend JC Middelkoop

**Affiliations:** 1 Department of Youth Health Care, Municipal Health Service The Hague (GGD Den Haag), Koningin Sophiestraat 120, 2595 TM The Hague, the Netherlands; 2 Department of Child Health, Netherlands Organisation for Applied Scientific Research TNO, Leiden, the Netherlands; 3 Department of Life Style, Netherlands Organisation for Applied Scientific Research TNO, Leiden, the Netherlands; 4 Department of Methodology & Statistics, Faculty of Social Sciences, University of Utrecht, Utrecht, the Netherlands; 5 Department of Epidemiology, Municipal Health Service The Hague (GGD Den Haag), The Hague, the Netherlands; 6 Department of Public Health and Primary Care, Leiden University Medical Center, Leiden, the Netherlands

**Keywords:** Birth weight, Infant, low birth weight, Infant, small for gestational age, India, The Netherlands

## Abstract

**Background:**

South Asian babies born in developed countries are generally lighter than babies from other ethnic groups born in the same country. While the mean birth weight of Caucasian babies in the Netherlands has increased the past decades, it is unknown if the mean birth weight of South Asian babies born in the Netherlands has increased or if the prevalence of low birth weight (LBW) or small-for-gestational-age (SGA) has decreased.

The aims of this study are: 1. to investigate secular changes in mean birth weight and the prevalence of LBW and SGA in Surinamese South Asian babies, and 2. to assess differences between Surinamese South Asian and Dutch Caucasian neonates born 2006–2009.

**Methods:**

A population based study for which neonatal characteristics of 2014 Surinamese South Asian babies, born between 1974 and 2009 in the Netherlands, and 3104 Dutch Caucasian babies born 2006–2009 were obtained from well-baby clinic records. LBW was defined as a birth weight <2500 g. SGA was based on a universal population standard (the Netherlands) and three ethnic specific standards (the Netherlands, UK, Canada).

**Results:**

In Surinamese South Asian babies from 1974 to 2009 no secular trend in mean birth weight and prevalence of LBW was found, whereas SGA prevalence decreased significantly.

Surinamese South Asian babies born in 2006–2009 (2993 g; 95% CI 2959-3029 g) were 450 g lighter than Dutch Caucasian babies (3448 g; 95% CI 3429-3468 g), while LBW and SGA prevalences, based on universal standards, were three times higher. Application of ethnic specific standards from the Netherlands and the UK yielded SGA rates in Surinamese South Asian babies that were similar to Dutch. There were considerable differences between the standards used.

**Conclusion:**

Since 1974, although the mean birth weight of Surinamese South Asian babies remained unchanged, they gained a healthier weight for their gestational age.

## Background

Birth weight is generally used as an indicator of a newborn’s wellbeing, and as an indirect measure of the intrauterine environment and the nutritional status of the mother during pregnancy. In developing regions with lower socioeconomic status and poorer nutrition, babies are lighter and more frequently have adverse birth outcomes compared to developed regions [1]. South Asia has the highest incidence of low birth weight (LBW, <2500 g) in the world (21-28%) [2] and one of the highest perinatal mortality rates [1]. Despite higher socioeconomic status and better nutrition, immigrant South Asian babies born in developed countries also tend to be lighter, shorter, and leaner at birth, and have a higher prevalence of LBW than their native counterparts [3-5]. However, there is increasing evidence that the lower birth weight and high rates of LBW and small-for-gestational-age (SGA, birth weight <10^th^ percentile for gestational age) in these populations are not expressions of fetal growth restriction, but are rather physiological or constitutional in origin [6-8]. Consequently, using a single population standard for determining SGA in South Asian babies is likely to cause misclassification of many healthy South Asian babies as SGA. For that reason, in several countries South Asian specific birth weight standards were developed, which demonstrated a much higher association between SGA and adverse birth outcomes than the single population standard [7,9,10].

In the Netherlands, perinatal mortality generally declined between 2000 and 2006 [11], but the rates in South Asian babies have remained considerably higher than in Dutch Caucasian babies [12]. It is unknown if this discrepancy is related to differences in birth weight or SGA prevalence. While the mean birth weight of neonates born in the Netherlands increased from 3372 grams in 1989 to 3466 grams in the years 2008–2010 [13], it is unknown if the mean birth weight of South Asian babies born in the Netherlands has increased, or if the prevalence of LBW or SGA has decreased.

The objectives of our study were firstly to determine if there are secular trends in birth weight and prevalence of LBW and SGA (based on universal and ethnic specific standards) in Surinamese South Asian babies born in the Netherlands, and secondly, to assess the differences in neonatal characteristics between Surinamese South Asian and Dutch babies born in 2006–2009, particularly the distributions of birth weight in both populations.

## Methods

### Data source

In the Netherlands, the health of all infants is periodically assessed by physicians and nurses of Youth Health Care at well-baby clinics. The results of these check-ups are registered in health records which are kept at least 10 years after the child’s 19^th^ birthday.

The records of all Surinamese South Asian children born in the periods 1974–1976 and 1991–1993 were analysed in a previous study [14]. For this current study all records of Dutch and Surinamese South Asian children born 2006–2009 were added. The following data were extracted from the records: the child’s family name, date of birth, sex, gestational age, birth weight, singleton or multiple birth, country of birth, and parity, together with the family name and country of birth of the mother and father.

As this study encompassed routinely collected data from medical records, under Dutch law approval by an ethical review committee was not needed [15]. Approval of the study protocol and permission to use the data for this study were obtained from the head of the department of Youth Health Care and from the head of the department of Epidemiology of the Municipal Health Service of the city of The Hague.

### Population

Most South Asian people in the Netherlands are descendants of Asian Indians who migrated from 1873 to 1916 to the former Dutch colony Suriname. At around the time of Suriname’s independence in 1975 many Surinamese South Asians moved to the Netherlands, to the city of The Hague in particular [16]. As Suriname is a multi-ethnic society, with people originating from the Netherlands, India, West-Africa, Java and China, the country of birth is insufficient to determine ethnicity. Therefore, the child’s ethnicity was defined by country of birth of both the father and mother, together with their respective family names.

Surinamese South Asian ethnicity was then determined by a typical Surinamese South Asian family name of both parents (by matching the names with a list of common Surinamese South Asian family names), and Suriname or the Netherlands as country of birth for respectively first generation and second generation parents in the Netherlands. Parents with a typical Dutch family name who were also born in the Netherlands were considered Dutch Caucasian. Only children of whom both parents were Surinamese South Asian or Dutch Caucasian were included in the study. In cases with one or both parental family names missing (4.3%), ethnicity was determined by the child’s family name and the available parental family name, together with the parent’s country of birth.

Based on these selection criteria, 2858 records of Surinamese South Asian children born in 1974–1976, 1991–1993 or 2006–2009, and 3256 records of Dutch children born 2006–2009 were retrieved. For the final selection, children born outside the Netherlands (n = 635) were excluded, as in these cases gestational age and birth weight were self-reported and therefore considered less reliable. Additionally, children of multiple birth (n = 92), or with a missing record of birth weight (n = 174) or gestational age (n = 95) were excluded. A total of 2014 records of Surinamese South Asian children and 3104 records of Dutch children remained for the analyses.

### Measurements and fetal growth standards

In all time periods a midwife or an obstetrician calculated the gestational age based on the first day of the last menstrual period. In cases with unknown or dubious last menstrual period, gestational age was determined from an ultrasound dating scan. The gestational age, together with other details regarding pregnancy and birth, were added to the child’s health record after a home visit by a Youth Health Care nurse in the second week postpartum. In 92% of cases of cohort 1974–1976 gestational age was recorded in completed weeks, and in the remainder in weeks and days. In 1991–1993 and 2006–2009 gestational age was registered in weeks and days. A gestational age of <37 weeks was considered a preterm birth. LBW was defined as a birth weight <2500 g. To estimate the prevalence of SGA in Dutch Caucasian and Surinamese South Asian babies, the most recent universal Dutch standard for birth weight by gestational age (gestational ages ≥25 and ≤42 weeks, sex and parity dependent) [17] was applied. Secondly, we determined SGA rates based on separate standards for babies of South Asian descent and for babies of European descent, from the Netherlands [17,18], the United Kingdom [19], and Canada [20]. A birth weight below the 10^th^ percentile for gestational age was defined as SGA. Children of unknown parity were defined as primiparous.

### Statistical analyses

As this study had two objectives, the data were analysed accordingly. Firstly, the data of Surinamese South Asian babies were analysed to assess the differences between the time periods, and secondly, differences between Dutch and Surinamese South Asian babies born in 2006–2009 were analysed.

Furthermore, we used a stepwise approach. In the first step we compared neonatal and maternal characteristics using Pearson’s Chi-square tests (categorical variables) and analysis of variance (mean gestational age). In the second step, birth weight characteristics were examined. To test for differences between birth weights, a general linear model (GLM) was used with birth weight as the dependent variable and sex, parity, and gestational age as adjusting factors. Differences in the prevalence of LBW (LBW vs. not LBW) and SGA (SGA vs. not SGA) were assessed with logistic regression analyses, firstly with time period as the independent variable to test for differences between Surinamese South Asian time periods and secondly with ethnic group as the independent variable to test for differences between Surinamese South Asian and Dutch babies born in 2006–2009. Analyses of LBW were adjusted for sex, parity, and gestational age. Analyses of SGA based on the South Asian standard from the Netherlands were adjusted for sex and parity, as this standard isn’t sex or parity specific. Analyses based on the sex-specific Canadian and British standard were adjusted for parity.

All analyses were conducted using IBM SPSS Statistics 20.

## Results

We found differences in neonatal and maternal characteristics between Surinamese South Asian cohorts, and between the Dutch and Surinamese South Asian population (Table 1). Within the Surinamese South Asian population, Surinam as the maternal birth country of birth declined from 100% in 1974–1976 to 67.3% in 2006–2009. The mean gestational age decreased significantly from 39.4 weeks in 1974–1976 to 39.0 weeks in 2006–2009. The distribution of gestational ages generally shifted to the left while the standard deviation increased. The mean gestational age was significantly shorter in the Surinamese South Asian cohort 2006–2009 than in Dutch Caucasian neonates, while preterm rates were twice as high.

**Table 1:** Child and maternal characteristics of Surinamese South Asian and Dutch population

		**Surinamese South Asian**			**Dutch**
		**1974-1976**	**1991-1993**	**2006-2009**	**2006-2009**
Number of births		337	830	847	3104
% Maternal country of birth^a^	Surinam	100	99.3	67.3	0
	The Netherlands	0	0.7	32.7	100
% Parity^c^	1	46.9	44.1	46.6	56.8
	>1	51.0	55.7	49.5	39.8
	Unknown	2.1	0.2	3.9	3.5
% Sex	Boy	49.6	50.2	51.6	50.8
	Girl	50.4	49.8	48.4	49.2
Gestational age, mean in weeks (SD)^b^		39.4 (1.7)	39.2 (1.9)	39.0 (2.0)	39.7 (1.7)
Gestational age categories^b^	<31	0.3	0.6	0.9	0.5
	31-32	0.6	0.6	1.2	0.4
	33-34	0.3	1.6	2.2	0.8
	35-36	4.7	4.9	6.1	3.8
	37-38	21.1	25.5	31.3	20.7
	39-40	57.3	51.1	45.3	50.7
	41-43	15.7	15.7	12.9	23.1
% Preterm, <37 weeks^b^		5.9	7.7	10.5	5.4

^a^significantly different 1. between Surinamese South Asian time periods, P < 0.05 and 2. between Dutch and Surinamese South Asian babies born 2006–2009, P < 0.001.

^b^significantly different 1. between Surinamese South Asian time periods, P < 0.01 and 2. between Dutch and Surinamese South Asian babies born 2006–2009, P < 0.001.

^c^Analysed without ‘unknown’category; Dutch significantly different from Surinamese South Asian 2006–2009, P < 0.001.

We found no secular changes in mean birth weight of Surinamese South Asian neonates (Table 2). LBW prevalence in Surinamese South Asian newborns showed a non-significant increasing trend since 1974–1976. Concurrently, the prevalence of SGA significantly decreased when both the Dutch universal standard, and the ethnic specific Dutch and British standards were applied. The British standard resulted in the lowest prevalence of SGA, whereas the universal Dutch and the Canadian standard yielded the highest prevalence.

**Table 2:** Birth weight characteristics with 95% confidence intervals (CI) of Surinamese South Asian and Dutch population

		**Surinamese South Asian**						**Dutch**	
		**1974-1976**		**1991-1993**		**2006-2009**		**2006-2009**	
		**Mean**	**95% CI**	**Mean**	**95% CI**	**Mean**	**95% CI**	**Mean**	**95% CI**
Birth weight in g									
	Unadjusted	2995	2941-3049	3038	3002-3074	2993	2958-3029	3448*^b^	3429-3468
	Adjusted^a^	2983	2878-3087	3067	2953-3181	3032	2982-3083	3461*^b^	3432-3489
Birth weight ≥37 wk in g									
	Unadjusted	3048	2998-3098	3113	3080-3145	3090	3060-3120	3505*^b^	3488-3523
	Adjusted^a^	3106	2988-3223	3144	3031-3258	3126	3070-3183	3517*^b^	3488-3547
% LBW									
	<2500 g	11.3	7.9-14.7	12.7	9.3-16.1	14.8	11.4-18.2	4.4*^b^	3.7-5.1

*P < 0.001.

^a^adjusted for sex, parity, and gestational age.

^b^Dutch significantly different from Surinamese South Asian 2006–2009.

Surinamese South Asian neonates born in 2006–2009 differed from Dutch babies in almost all birth weight characteristics (Table 1). The mean birth weight was around 450 g lower than in Dutch babies.

**Figure 1:**
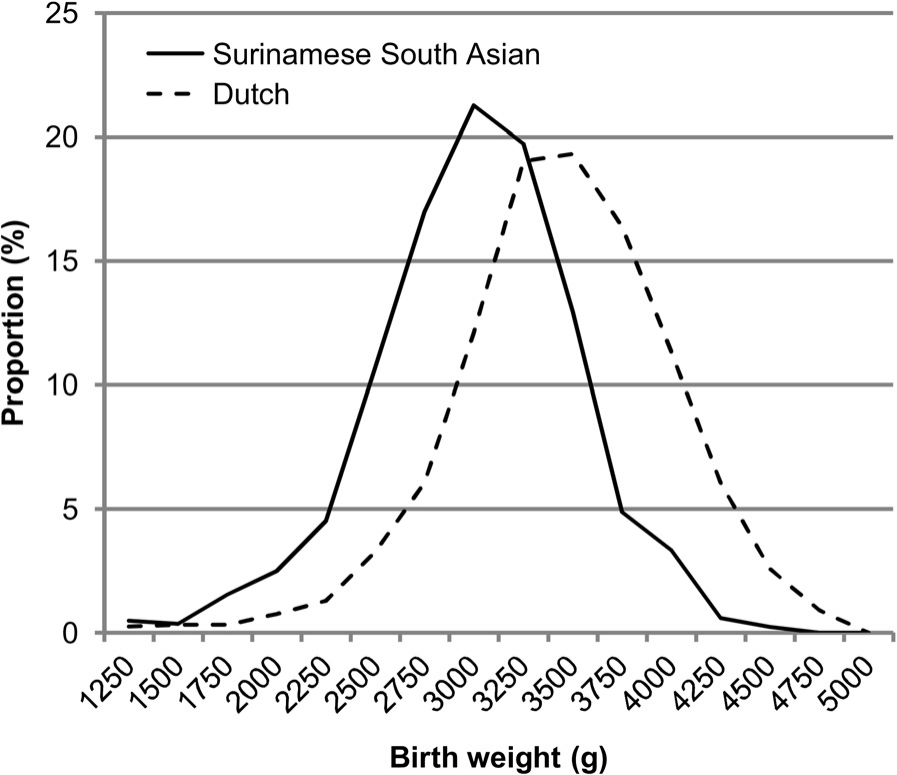
Birth weight distribution of Surinamese South Asian and Dutch neonates, born 2006–2009.

**Figure 2:**
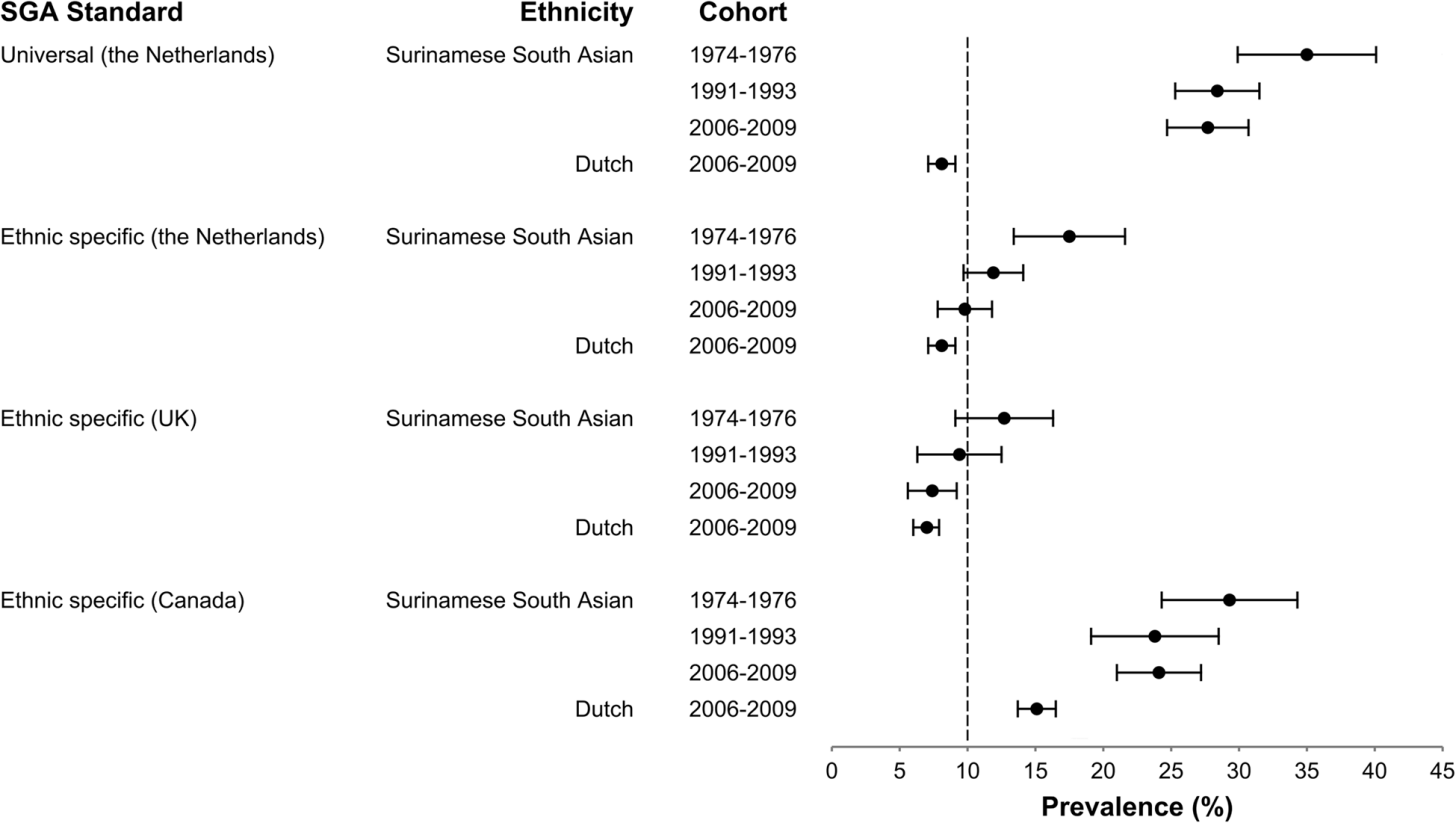
**Prevalence of SGA with 95% confidence interval, by SGA standard in Surinamese South Asian and Dutch neonates.** The dashed vertical line at the 10 percent mark represents the reference value of the SGA standards.

While the shape and spread of both distributions were similar (Figure 1), the Surinamese South Asian curve (mean ± SD: 2993 ± 521) was shifted to the left relative to the Dutch curve (mean ± SD: 3448 ± 552). As a result, the proportion of LBW <2500 g neonates was over three times higher in Surinamese South Asian babies (Table 2). SGA prevalence, based on the universal Dutch standard, was also more than three times higher in Surinamese South Asian babies (Figure 2). The application of the ethnic specific Dutch and British standards resulted in similar rates of SGA in Dutch and Surinamese South Asian babies. However, using the Canadian standard resulted in SGA rates that were higher than expected, both in Dutch and Surinamese South Asian babies.

## Discussion

This study shows that the mean birth weight of Surinamese South Asian neonates did not significantly change over a period of 35 years. As the mean birth weight of Dutch babies was previously shown to have increased by almost 100 grams since the late 1980′s [13], this finding implies that the difference in mean birth weight between Surinamese South Asian and Dutch neonates has become greater.

However, mean birth weight alone does not tell the whole story. The decrease in SGA rates in our population showed that Surinamese South Asian neonates have actually gained weight, but this effect was not reflected in higher mean birth weights, most likely due to a considerable increase in the prevalence of preterm delivered babies. Contrary to reports from other countries which found increasing trends in the proportion of preterm delivered babies [21], preterm rates in the Netherlands have decreased in the past decade [22]. The cause of preterm rates in the Surinamese South Asian population in the Netherlands increasing strongly since 1974 is unclear. It could be explained by a generally higher prevalence of pathology during pregnancy in South Asian women, such as pre-eclampsia and gestational diabetes [23], leading to earlier complications during pregnancy and thus subsequently a relatively higher proportion of induced labour. For our study, information on the proportion of such iatrogenic preterm deliveries was not available.

The strengths of this study are the large sample size, and the availability of some important confounding factors. However, not all known confounders such as maternal age, socio-economic status, maternal height and pre-pregnancy weight, and information on smoking during pregnancy were available to enable adjustment. Another limitation is that a change in pregnancy dating methods, from menstrual dating in the early years to menstrual and ultrasound dating in the more recent years, could have influenced the temporal trends among the Surinamese South Asian infants. Lastly, children with congenital or chromosomal anomalies could not be excluded, as this information was unavailable. Nevertheless, as most of these children receive specialised care and do not usually attend the standard check-ups at well-baby clinics, it is expected that only a small proportion of these children were included in our study.

Compared with the birth weights of South Asian babies in other countries, the mean birth weight of Surinamese South Asian babies born in 2006–2009 (3032 g) was lower than the birth weights of South Asian babies in the United States (3170 g) [3], Canada (3221 g) [24], Norway (3244 g) [10], and the UK (3072-3129 g) [4]. This difference may be the result of our stricter selection criterion for ethnicity. In our study both the country of birth and the family name of both parents were required, whereas in many other studies only the background data of the mother were used, which may have led to a less homogeneous group.

In addition, there may also be differences between the populations of South Asians in these countries. For example, the Norwegian study [10] only included babies with a Pakistani ethnicity. In Britain, Pakistani (3129 g) babies were also shown to be heavier than babies of Bangladeshi (3072 g) or Asian Indian (3087 g) parents [4]. Therefore, the term ‘South Asian’ does not entail homogeneity. This is also reflected in differences between the birth weight standards from these countries, with the Canadian standard yielding considerably higher SGA rates than the ethnic specific Dutch and the British standard.

While the large left shift of the birth weight distribution of Surinamese South Asian babies compared with that of Dutch Caucasian babies was remarkable, similar differences in birth weight between South Asian and Caucasian neonates have been observed in other developed countries [3,6,7]. The differences have been attributed to physiological variations in body composition, which in South Asian babies is expressed as a smaller muscle mass but similar fat stores compared with Caucasian babies [25]. To account for such ethnic variation and other physiological factors in the assessment of the nutritional status of neonates, ethnic specific or customised fetal growth standards are recommended for use in clinical practice. The application of such standards generally improves the prediction of adverse birth outcomes [7,9,10,26].

## Conclusion

In conclusion, this study found no secular changes in mean birth weight and LBW prevalence in Surinamese South Asian babies since 1974. While not expressed in the mean birth weight, SGA prevalence decreased significantly, indicating that Surinamese South Asian babies have actually gained a healthier weight for their gestational age. Surinamese South Asian babies were approximately 450 grams lighter than Dutch Caucasian neonates, and LBW and SGA was highly prevalent when based on a single standard. When applying ethnic specific criteria to determine SGA, the rates were concordant with those found in Dutch Caucasian babies.

## Competing interests

The authors declare that they have no competing interests.

## Authors’ contributions

JAdW designed the study, extracted, analysed and interpreted the data and wrote the first draft of the manuscript. SvB participated in the analyses, interpretation of the results and revision of the manuscript. BJCM contributed to the design of the study, helped with the interpretation of the results and revising the manuscript. All authors read and approved the final version of the manuscript.

## Pre-publication history

The pre-publication history for this paper can be accessed here:

http://www.biomedcentral.com/1471-2458/13/931/prepub
